# A new nonsense mutation of PTCH1 gene in mother and daughter with late-onset nevus basal cell carcinoma syndrome: Case report

**DOI:** 10.1097/MD.0000000000040471

**Published:** 2024-11-29

**Authors:** Xin Li, Liya Ai, Chun-Yu Han, Ya-Qi Cao, Jian-Wen Han

**Affiliations:** a Department of Dermatology, The Affiliated Hospital of Inner Mongolia Medical University, Hohhot, China.

**Keywords:** nevoid basal cell carcinoma syndrome, nonsense mutation, PTCH1

## Abstract

**Rationale:**

Nevoid basal cell carcinoma syndrome (NBCCS) is a rare clinical disease characterized by a disproportionate number of basal cell carcinoma to sun exposure and skin types. Patched 1 (PTCH1) gene is proposed to be implicated in the pathogenesis of NBCCS. This study aimed to investigate whether PTCH1 gene is the causative gene in Chinese patients with NBCCS.

**Patient concerns:**

Here we detected the first nonsense mutation in PTCH1 gene by Sanger sequencing of blood samples from a mother and her second daughter (NM000264: exon14: c.2080C>T: p.Q694X).

**Diagnoses:**

Both of the mother and her second daughter had ovarian mature teratomas.

**Interventions:**

The mother received liquid nitrogen cryotherapy, surgical resection, and radiation therapy, while her second daughter was treated with a GX-III multifunctional ion surgical therapy machine and surgery.

**Outcomes:**

New rashes continued to appear and contractures of the right eyelid healed in the mother, while her second daughter had multiple pitting depressions on the palms and soles of both hands and feet.

**Conclusion:**

We detected a new mutation in PTCH1 gene in 2 patients with NBCCS, and both of them had ovarian mature teratomas, which are related to NM000264: exon14: c.2080C>T: p.Q694X.

## 1. Introduction

Nevoid Basal Cell Carcinoma Syndrome (NBCCS), also known as Gorlin syndrome, is a rare autosomal dominant disorder that often develops in young adulthood and presents with basal cell carcinoma of the skin inconsistent with age, sun exposure, and the number of skin types.^[1]^

Patched 1 (PTCH1) gene is proposed to be implicated in the pathogenesis of NBCCS. PTCH1 gene is located at 9q22.32 and contains a total of 28 exons, which encodes a large transmembrane protein that can regulate the hedgehog signaling pathway. There are 5 hedgehogs in nature, of which sonic hedgehogs (SHHs) are widely distributed in the human body and play a key role in human growth and development and tumor formation. PTCH is a receptor for SHH, which binds to Ptch or Ptch-Smo (active smoothed protein) complexes. In the absence of Ptch, Smo is activated and provides mitotic or differentiation signals to basal cells in the skin to cause NBCCS or skin basal cell carcinoma.^[2]^

To investigate whether PTCH1 gene is the causative gene in Chinese patients with NBCCS, in this study we detected PTCH1 gene mutation by sequencing analysis in the mother and her second daughter presented with NBCCS.

## 2. Case presentation

**Figure 1.**
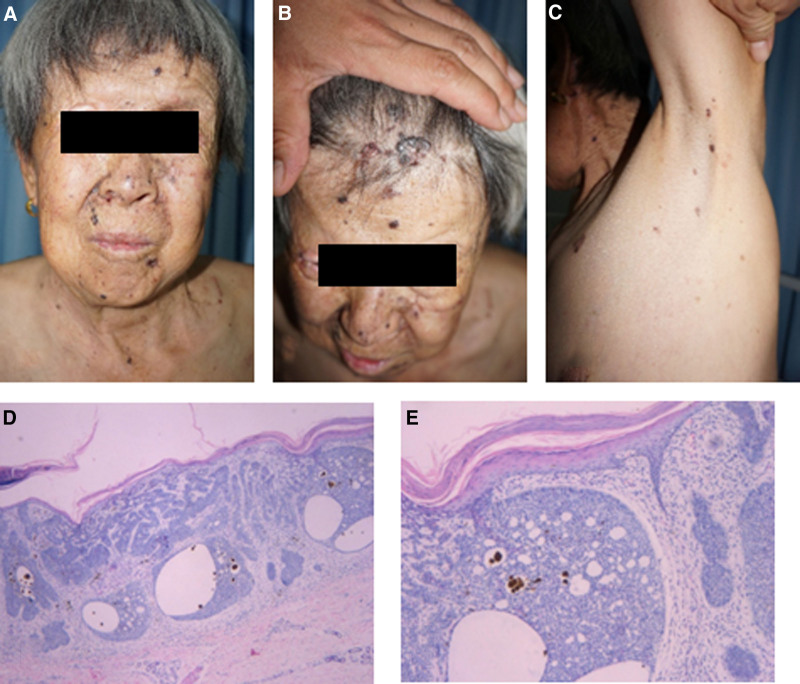
The proband had multiple nodules and ulcers on the face (A), the head (B) and the trunk (C). Pathological analysis showed hyperkeratosis, parakeratosis, thinning of the epidermis, basal-like cell tumor borders in the superficial layer of the dermis, arranged in a palisade shape. (D) (×40), (E) (×200).

The proband was a 71-year-old female presented with recurrent ulceration of facial brown rash with pain for 30 years. During the past 30 years, nodules and ulcers appeared successively on the cheeks, forehead, periocular region, trunk, and groin, and skin pathology revealed basal cell carcinoma of the skin (Fig. 1A–E). Although she received liquid nitrogen cryotherapy, surgical resection, and radiation therapy, new rashes continued to appear and contractures of the right eyelid healed. Past medical history: “mature teratoma of the ovary” was diagnosed 8 years ago; and “cataract enucleation of left eye” was diagnosed 10 years ago.

**Figure 2.**
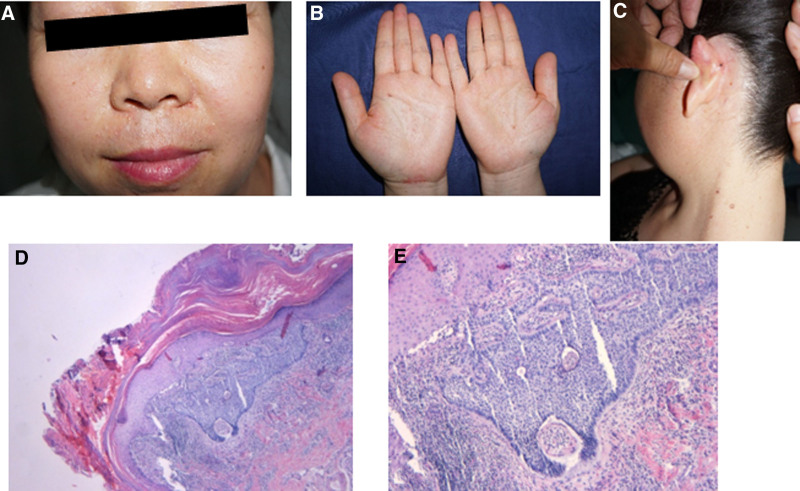
Multiple nodules on the face (A) and neck (C) of the proband’s second daughter with punctate depressions on the palms (B). Pathological analysis showed high degree of hyperkeratosis, parakeratosis, massive serovar scabs, irregular thickening of epidermis, basal-like cell mass in superficial dermis with palisade-like borders and gaps. (D) (×40), (E) (×200).

The proband underwent head and neck magnetic resonance imaging: combined with medical history, basal cell carcinoma of the left cheek and periauricular region was considered. Chest X-ray and abdominal ultrasound were normal; blood samples for blood routine, blood biochemistry, thyroid and parathyroid function, immune parameters and tumor markers and other tests were normal. The proband’s second daughter was 42 years old, and had dozens of black papules and nodules on the face, neck, trunk, and both upper limbs since the age of 30 years (Fig. 2A–C). Skin pathological biopsies revealed basal cell carcinoma of the skin (Fig. 2D–E). She was treated with a GX-III multifunctional ion surgical therapy machine and surgery, and now has multiple pitting depressions on the palms and soles of both hands and feet. Past medical history: Ovarian mature teratoma was diagnosed at the age of 34 years. The proband’s eldest daughter, 51 years old, had a similar rash on her face, neck, and trunk, and no detailed clinical information and blood samples were collected as she lived in a different place.

**Figure 3.**
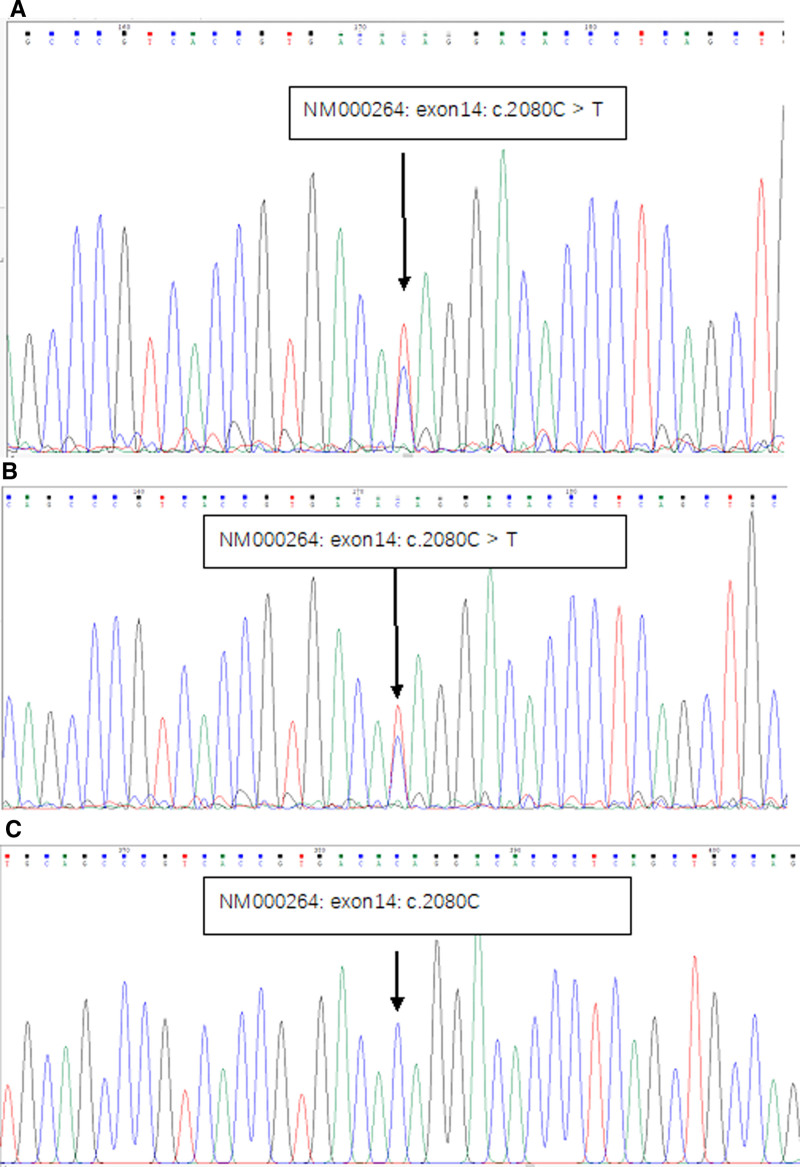
Sanger sequencing of PTCH1 gene. (A) proband, (B) proband second daughter, and (C) healthy controls.

Sanger sequencing analysis was performed based on the peripheral blood samples of the patient and her second daughter, with the forward primer sequence (5′–3′): GTTGTGGCAGATTACCTTGGC; and reverse primer sequence (5′–3′): AGGCGATGAACCAGGTGATG. The fragment was 601 bp in total, and a nonsense mutation (c.2080C>T) at nucleotide C at position 2080 in exon 14 of the PTCH1 gene was detected (Fig. 3), resulting in the mutation of the glutamine at position 543 to a stop codon due to the change in the above base, which prematurely terminated peptide chain synthesis (p.Q543X).

## 3. Discussion

NBCCS is a rare disease with variable expression and nearly complete penetrance. According to the latest diagnostic criteria of NBCCS,^[3]^ the mother and daughter could have NBCCS: dozens of basal cell carcinoma lesions, out of proportion to sun exposure and skin type; first-degree relatives with NBCCS, and they had PTCH1 gene mutation; the proband’s second daughter had punctate depressions on the palms and soles. In the process of differential diagnosis, NBCCS must be distinguished from other diseases exhibiting similar dermatological manifestations based on pathological biopsy. Malignant melanoma, for example, can present as multiple dark nodules, with pathological examination revealing infiltrative growth of spindle cells, pronounced nuclear division, and a high propensity for metastasis. Basosquamous carcinoma denotes a tumor containing both basal cell and squamous cell carcinoma components. Seborrheic keratosis may also manifest similar skin lesions, but it is a benign condition without heritability and typically lacks concurrent systemic symptoms. Furthermore, genetic testing is important to differentiate skin lesions arising from nongenetic mutations, such as multiple basal cell carcinomas induced by arsenic poisoning. This condition is characterized by a history of chronic arsenic exposure.

PTCH1 gene is involved in the pathogenesis of NBCCS.^[3]^ In this study, the proband and her second daughter had a nonsense mutation (NM000264: exon14: c.2080C>T: p.Q694X) at nucleotide C at exon 2080 of PTCH1 gene, leading to prematurely terminated peptide chain synthesis. However, this mutation was not detected in healthy controls. Review of the published NBCCS family whole exome sequencing and human gene mutation database showed that 89% of families had pathogenic PTCH1 mutations, mainly nonsense or frame shift mutations (64%), followed by splice site mutations (13%), insertions or deletions (12%) and missense mutations (8%).^[4,5]^ We checked OMIM and PuMed and the nonsense mutations carried by the 2 patients in this study were not reported before.

Nearly 100 loci of PTCH1 gene mutations were listed on the website of the Department of Molecular Genetics, Kitasato University, Japan, which were widely distributed on most exons, especially exon 2, 3, 9, and 19.^[6,7]^ Different mutations of PTCH1 may lead to different clinical manifestations in patients, and the more common manifestations are falx calcification, bone malformations, ocular abnormalities, cleft lip and palate and other diseases, with the vast majority of patients showing the manifestations before the age of 20 years.

However, the age of onset of the mother and the daughter in this case was late, and the proband developed the first rash at the age of 40 years and lacked the above common clinical symptoms. However, it is worth mentioning that both the mother and the daughter had a history of “mature teratoma of the ovary.” It is 1 limitation of this study that we did not investigate the association between c.2080C>T and ovarian mature teratoma and need further studies.

PTCH1 is the key component of Hedgehog (Hh) signaling pathway, which is involved in many physiological processes in the body.^[8]^ Recently, Hh pathway inhibitors vismodegib and sonidegib have been used in patients with basal cell carcinoma following surgery or radiation therapy.^[9,10]^ Another limitation of this study is that we did not evaluate the application of Hh pathway inhibitors in our patients.

In conclusion, a new mutation in the PTCH1 gene was detected in 2 Chinese patients with NBCCS in this study, and both of them had ovarian mature teratomas, which are related to NM000264: exon14: c.2080C>T: p.Q694X. Our findings will help reveal the pathogenesis of NBCCS and the link between NBCCS and ovarian mature teratomas.

## Acknowledgments

This study was supported by Inner Mongolia Science and Technology Plan (2019GG082) and Scientific and technological innovative research team for Inner Mongolia Medical University of “Basic and clinical research of psoriasis” (YKD2022TD030).

## Author contributions

**Conceptualization:** Jianwen Han.

**Investigation:** Xin Li, Liya Ai, Chunyu Han, Yaqi Cao.

**Supervision:** Jianwen Han.

**Writing – original draft:** Jianwen Han.

**Writing – review & editing:** Jianwen Han.
